# Supplementary material for: HGF/R-spondin1 rescues liver dysfunction through the induction of Lgr5+ liver stem cells
Source: Nat Commun. 2017 Oct 27;8:1175. doi: 10.1038/s41467-017-01341-6 (PMC5660090; doi:10.1038/s41467-017-01341-6)
Supplement: Supplementary file 1 — Supplementary Information [file 41467_2017_1341_MOESM1_ESM.pdf]

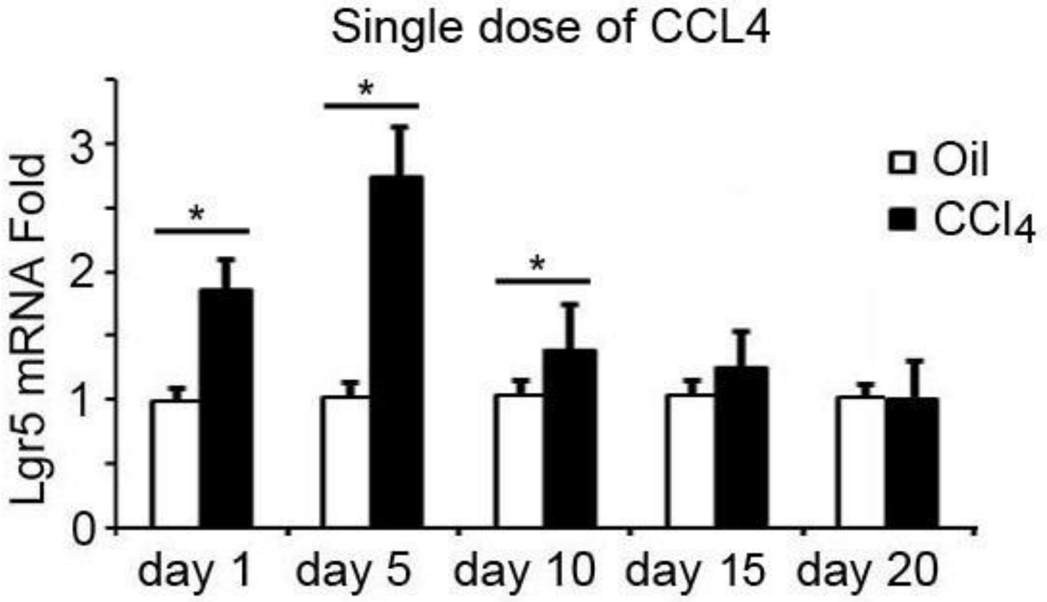


**Supplementary Figure 1: Lgr5 expression in liver was induced by CCL4 and decreased after liver recovery.** Mice were treated with single dose of CCL4 or control oil, the livers were collected at various time-points and the Lgr5 mRNA level were measured using qPCR assay. n= 5 mice per group. Triplicates for each condition were analysed. Results are shown as mean ± s.d. of three independent experiments. *, $p$<0.05.

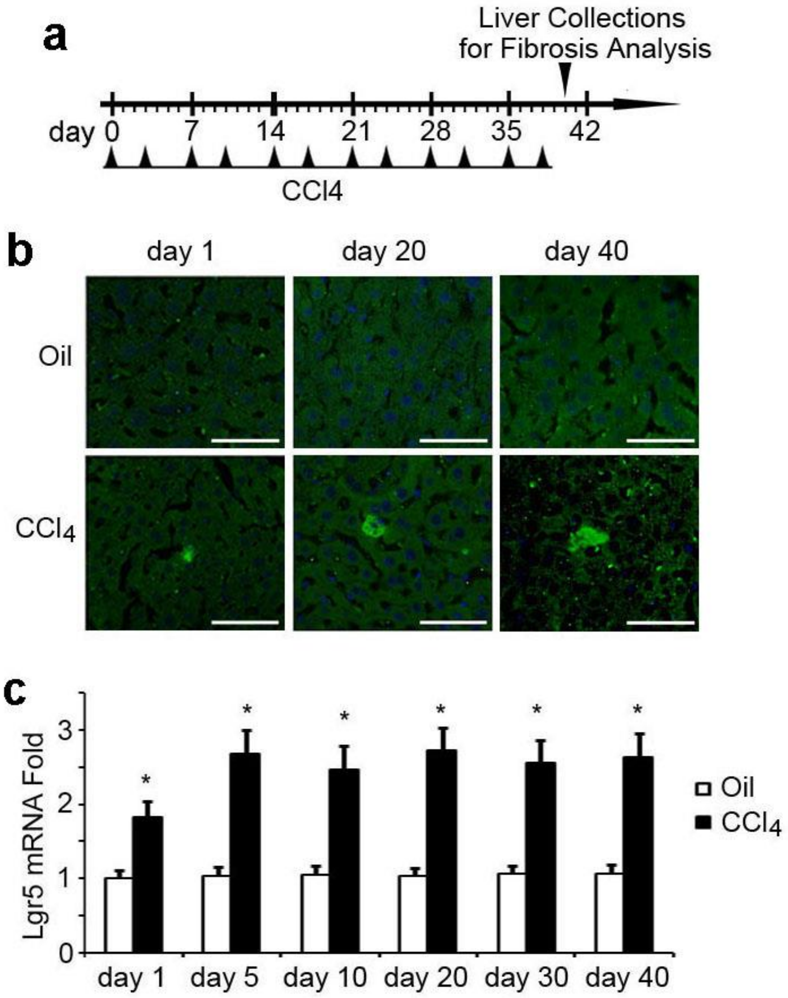


**Supplementary Figure 2: Lgr5$^{+}$ cells were induced during process of CCL4-induced liver fibrosis development. a,** Schematic overview of experimental setup. 8-week old Lgr5-GFP mice were i.p. injected with CCL4 (2ml/kg, Sigma-Aldrich) dissolved in olive oil at 1:4, or olive oil alone (2ml/kg) twice a week for 6 weeks. The Lgr5 expression were stained using anti-GFP antibody (**b**), the Lgr5 mRNA level were measured using qPCR assay (**c**). n= 3 mice/group. Triplicates for each condition were analysed. Results are shown as mean ± s.d. of three independent experiments. *, $p$<0.05. Scale bar, 200 μm.

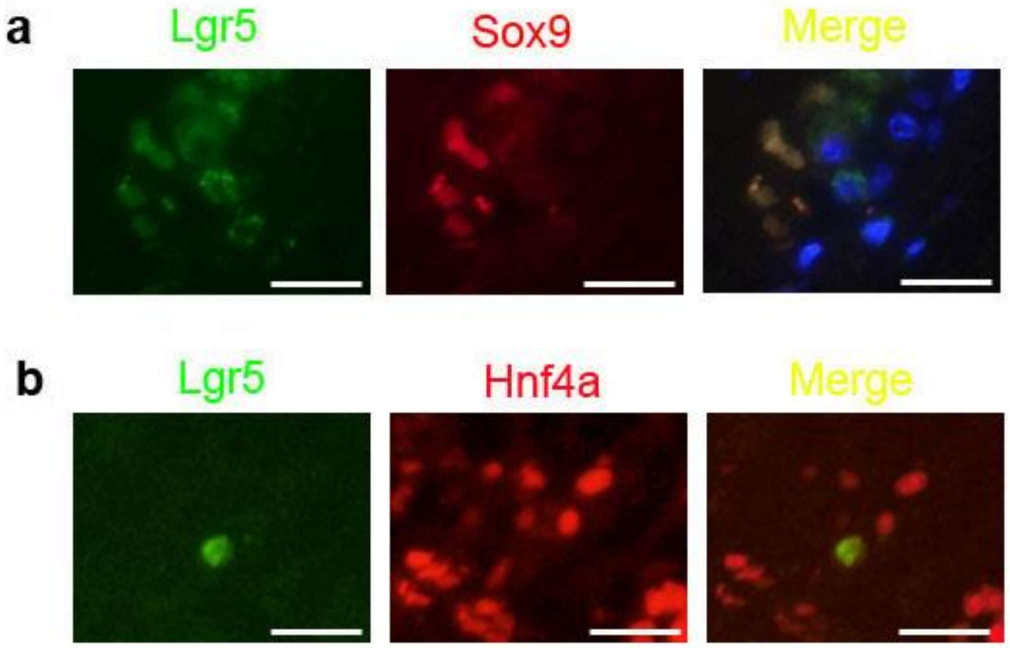


**Supplementary Figure 3: Expression of Sox9 and Hnf4a in Lgr5+ liver stem cells in liver fibrosis model.** 8-week old Lgr5-GFP mice were i.p. injected with CCL4 (2ml/kg, Sigma-Aldrich) dissolved in olive oil at 1:4, or olive oil alone (2ml/kg) twice a week for 6 weeks, the livers were collected for co-staining Lgr5 and Sox9 (**a**) or Lgr5 and Hnf4a (**b**). Results represent three separate experiments. Scale bar, 50 μm.

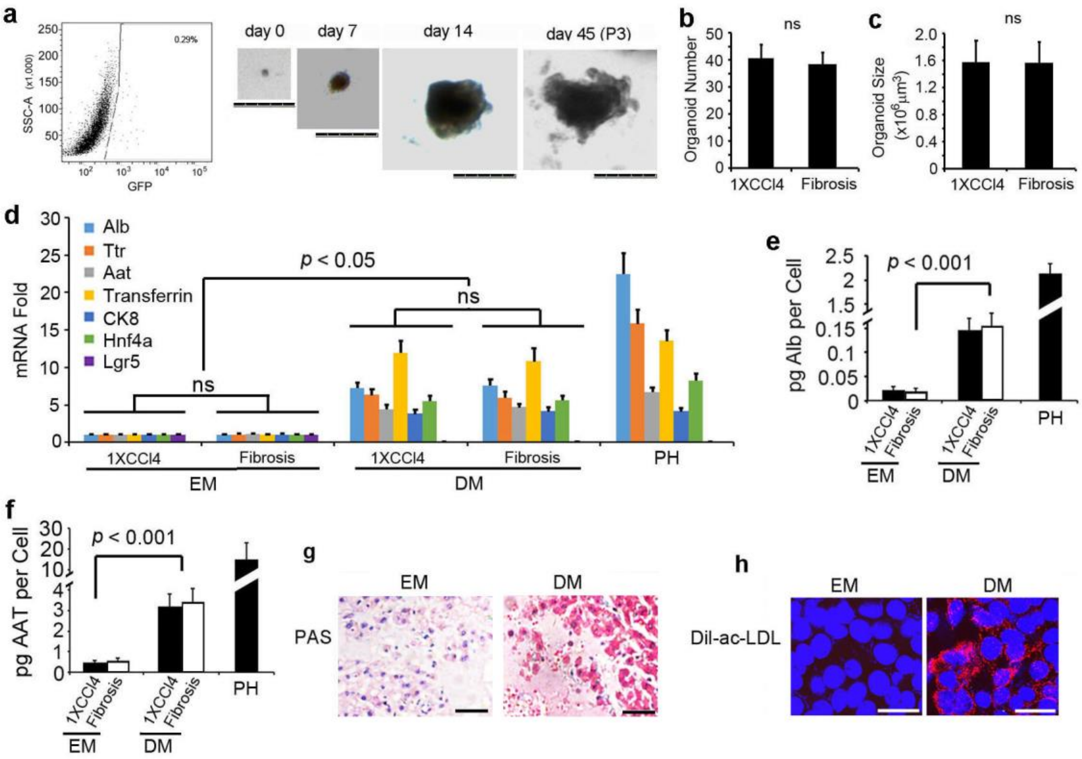


**Supplementary Figure 4: Lgr5+ liver stem cells induced upon chronic damage were similar to the ones induced upon 1XCCL4. a,** Lgr5-GFP mice were i.p. injected with CCL4 6 weeks as supplementary fig. 2a, Single Lgr5-GFP$^+$ cells were isolated on day 40 and cultured (FACS plots), serial images showing the outgrowth of a single Lgr5-GFP$^+$ cell. Scale bar, 250 μm. **b-f,** Lgr5-GFP$^+$ cells induced by 1XCCL4 on day 5 or chronic damage on day 40 as supplementary fig. 2a were analyzed. **b,c,** the organoids number (b) and organoids size (c) were analyzed. **d**. Hepatic gene expression in 1XCCL4 induced or chronic damage induced Lgr5-GFP$^+$ cells under expansion condition (EM) or differentiation condition (DM). **e, f,** Excretion of ALB (**e**) and ATT (**f**) in 1XCCL4 induced or chronic damage induced Lgr5-GFP$^+$ cells under expansion condition (EM) or differentiation condition (DM) by ELISA. **g**, Glycogen accumulation was determined by periodic acid-Schiff (PAS) staining in organoids grown in expansion medium or differentiation medium for 10 days. Scale bar, 100μm. **h**. Low-density lipoprotein (LDL) uptake was analysed using Dil-ac-LDL fluorescent substrate (red) in cultures mantained in expansion condition (EM) or differentiation condition (DM) for 14 days. Only cultures maintained in differentiation medium incorporated the substrate (red). Nuclei were counterstained with DAPI (blue). Scale bar, 50μm (**g, h**). For **e, f**, triplicates for each condition were analysed. Results are shown as mean ± s.d. of three independent experiments. For **b,** results are shown as mean ± s.d. of three independent experiments.

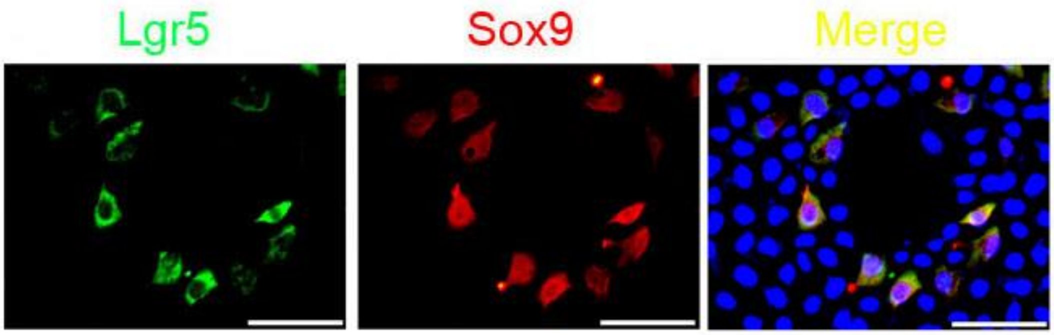


**Supplementary Figure 5: Expression of Sox9 in transplanted Lgr5$^+$ liver stem cells.** Lgr5-GFP$^+$ liver stem cells were isolated from *Lgr5-GFP* mice treated with 1XCCL4 by FACS, and these cells were injected intrasplenically into the wild type C57 mice with chronic damage (liver fibrosis model, 2XCCL4 treatment/week for 6 weeks, Fig. 2a). The livers were collected at day 40 for co-staining Lgr5 and Sox9. Results represent three separate experiments. Scale bar, 50 μm.

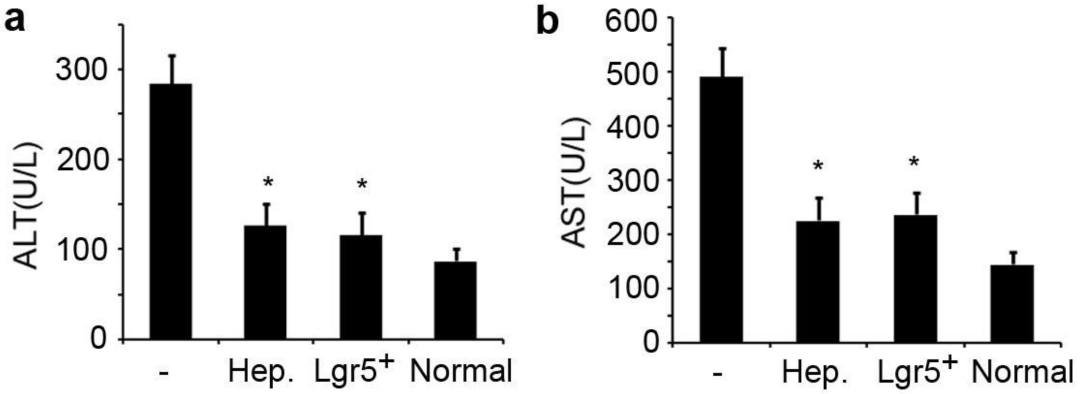


**Supplementary Figure 6: Lgr5+ liver stem cells transplantation reduced acute liver damage caused by single dose of CCL4.** 8-week old wild type C57 mice were i.p. injected with CCL4 (2ml/kg, Sigma-Aldrich) dissolved in olive oil at 1:4 for once. Lgr5-GFP$^+$ liver stem cells or primary hepatocyte (PH) derived from Lgr5-GFP mice were transplanted into the liver fibrosis mice by intrasplenically injection on day 0. The serum were harvested for ALT (a) and AST (b) analysis at day 2. n=5 mice/group. Triplicates for each condition were analysed. Results are shown as mean ± s.d. of three independent experiments. *, $p$<0.05.

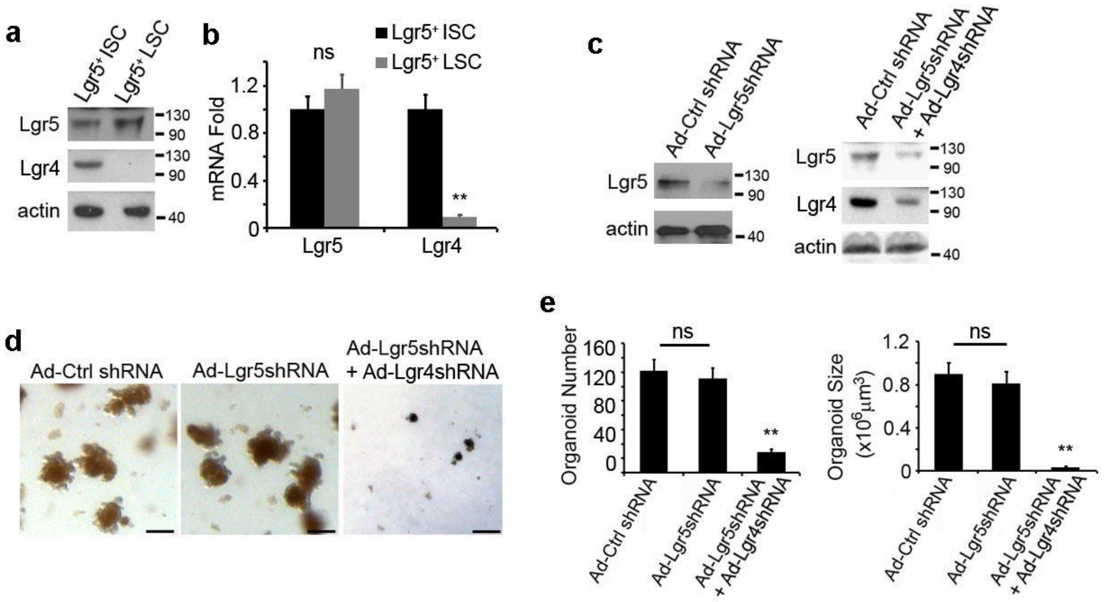


**Supplementary Figure 7: Liver Lgr5$^+$ stem cells did not express Lgr4.** Lgr5$^+$ intestine stem cells (Lgr5$^+$ ISC) were isolated from Lgr5-GFP mice, Lgr5$^+$ liver stem cells (Lgr5$^+$ LSC) were isolated from Lgr5-GFP mice upon chronic CCL4 damage as described in supplementary Figure 2a on day 40. **a**, The Lgr5 and Lgr4 expression in Lgr5$^+$ intestine stem cells (Lgr5$^+$ ISC) and Lgr5$^+$ liver stem cells (Lgr5$^+$ LSC) were stained using anti-Lgr5 and anti-Lgr4 antibody. **b**, the Lgr5 mRNA level in intestine stem cells (Lgr5$^+$ ISC) and Lgr5$^+$ liver stem cells (Lgr5$^+$ LSC) were measured using qPCR assay. **c**, Lgr5 were knocked down in Lgr5$^+$ liver stem cells by Ad-Lgr5shRNA (left), Lgr5 and Lgr4 were knocked down in Lgr5$^+$ intestine stem cells (right). **d, e,** Single Lgr5$^+$ intestine stem cells were cultured for organoid formation when treated with control none sense shRNA (Ad-Ctrl shRNA), Lgr5 shRNA alone (Ad-Lgr5shRNA) or Lgr5 shRNA plus Lgr4 shRNA (Ad-Lgr5shRNA+Lgr4shRNA). **d**, Represent organoid pictures. Scale bar, 200 μm. **e**, the number and sizes of intestine organoid were measured at day14. Results are shown as mean ± s.d. of three independent experiments. **, $p$<0.01.

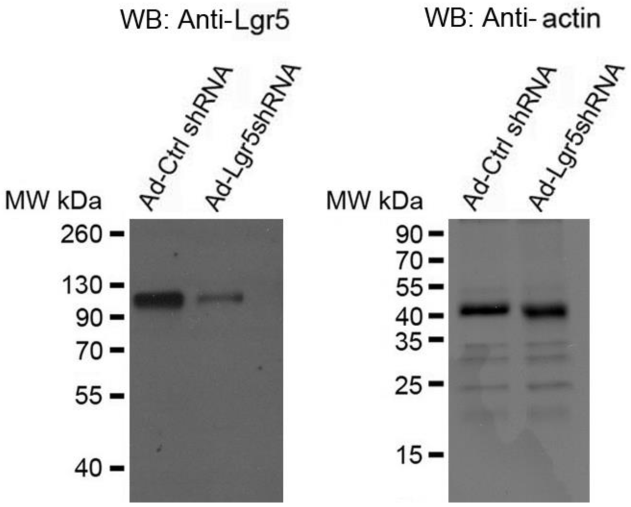


**Supplementary Figure 8:** Uncropped scans of western blots for Figure 2a. Results represent three or more separate experiments.

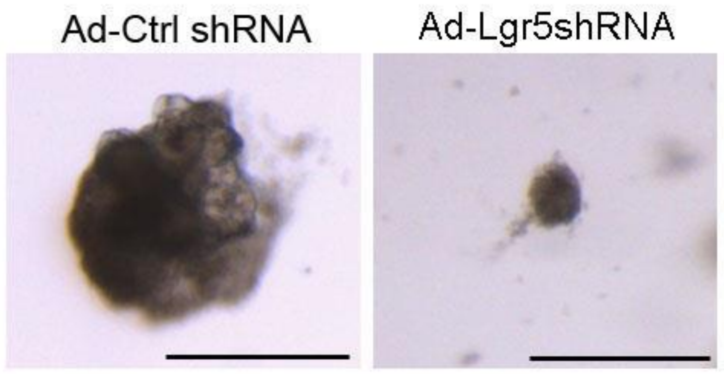


**Supplementary Figure 9: Enlarged images for cultured organoids.**

Sorted Lgr5$^{+}$ liver stem cells was infected with Ad-V or Ad-Lgr5-shRNA. They were cultured on Matrigel for 14 days. Results represent three or more separate experiments. Scale Bar, 200 $\mu$m.

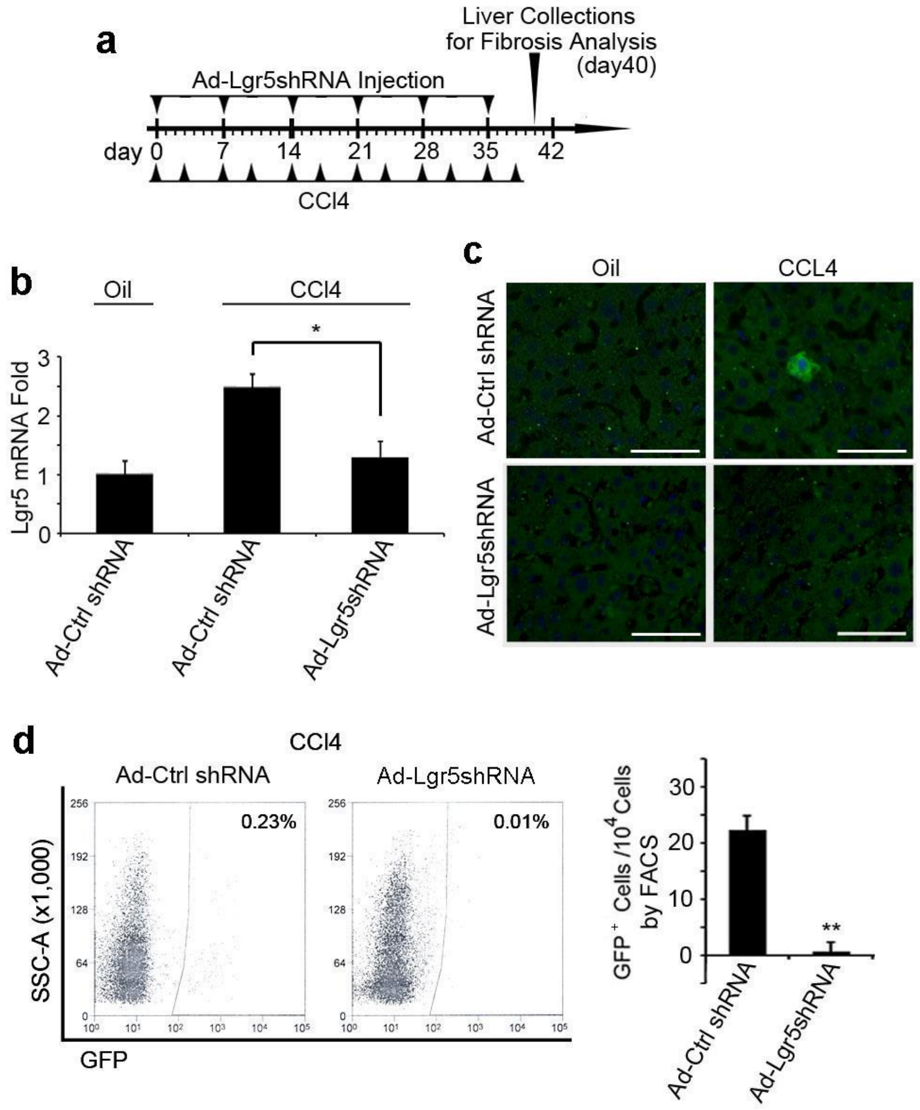


**Supplementary Figure 10: Ad-Lgr5shRNA down-regulated Lgr5 expression and Lgr5$^{+}$ cells number. a,** Schematic overview of experimental setup. 8-week old Lgr5-GFP mice were i.p. injected with CCL4 (2ml/kg, Sigma-Aldrich) dissolved in olive oil at 1:4, or olive oil alone (2ml/kg) twice a week for 6 weeks. Ad-Lgr5shRNA or Ad-Ctrl shRNA control were injected into mice tail once every week. The livers were harvested at day 40, the Lgr5 mRNA level were measured using qPCR assay (b), the Lgr5$^{+}$ cells were stained using anti-GFP antibody (c), the Lgr5$^{+}$ cells number were analyzed using FACS assay (d). n= 6 mice/group; for **b**, triplicates for each condition were analysed. Results are shown as mean ± s.d. of three independent experiments. *, $p$<0.05. For **d**, results are shown as mean ± s.d. of three independent experiments. **, $p$<0.01. Scale bar, 200 μm.

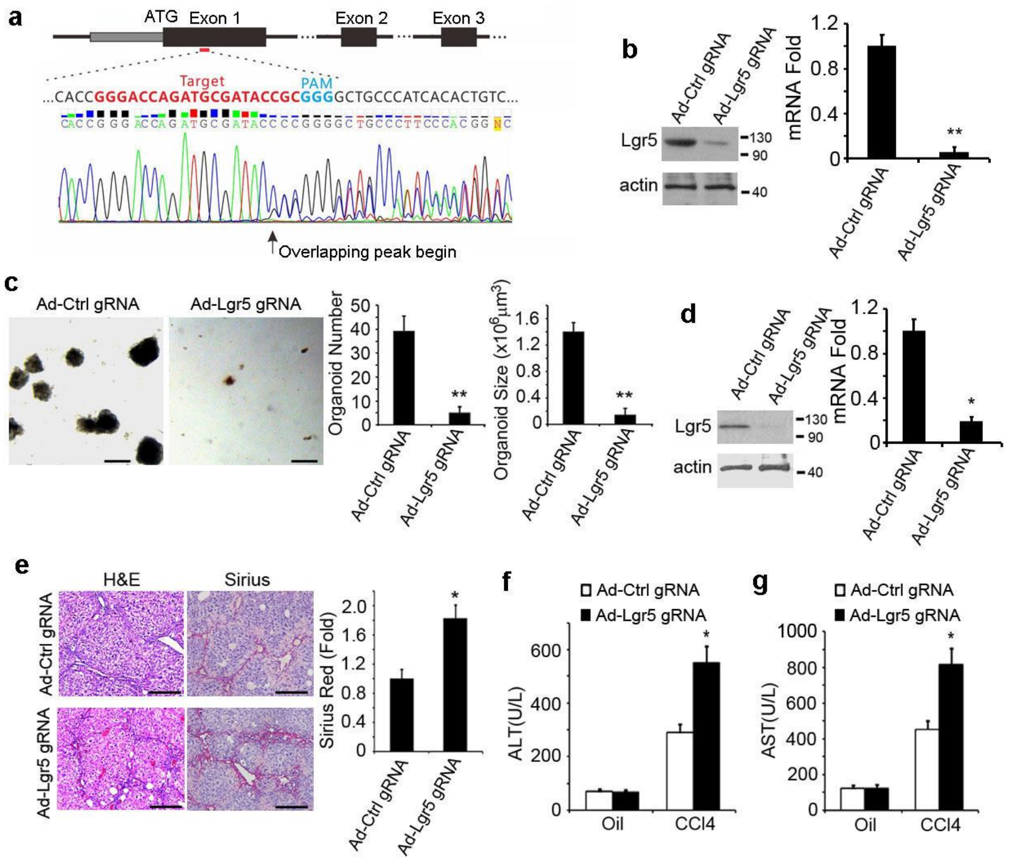


**Supplementary Figure 11: Knockout of Lgr5 using CRISPR-Cas9 blocked Lgr5$^+$ liver stem cells organoid formation ability *in vitro* and increased mice liver fibrosis *in vivo*. a,** Target site of gRNA and the result of direct sequencing. Overlapping peak mean that the CRISPER-Cas9 system worked well. **b**, The Lgr5 protein (left) and mRNA level (right) of Lgr5$^+$ liver stem cells infected with adenovirus encoding Cas9 plus Lgr5 gRNA (Ad-Lgr5 gRNA) or control none sense gRNA (Ad-Ctrl gRNA). **c**, Lgr5$^+$ liver stem cells failed to form organoid when Lgr5 was knocked out using CRISPER-Cas9 system. The representative organoids pictures (left), numbers (middle) and sizes (right) analysis. **d-g**, CCL4 induced C57 mice fibrosis model (as supplementary figure 2a) were infected with adenovirus encoding Cas9 and Lgr5 gRNA (Ad-Lgr5 gRNA) or control none sense gRNA (Ad-Ctrl gRNA) through i.v. every 7 days, the livers were collected and analyzed at day 40. **d**, Lgr5 protein (left) and mRNA level (right). **e**, Representative histology of H&E and Sirius Red and quantification of positive staining areas measured by Image J software. **f, g,** Serum levels of ALT (f) and AST (g) were measured. For **b, d, f, g**, triplicates for each condition were analysed. Results are shown as mean ± s.d. of three independent experiments. *, $p$<0.05**, $p$<0.01. For **c**, results are shown as mean ± s.d. of three independent experiments. **, $p$<0.01. For **e**, results are shown as mean ± s.d. of 5 independent sections taken randomly per mice and total 50 tissue specimens in each group (n=10 mice) *, $p$<0.05. Scale bar, 200 μm.

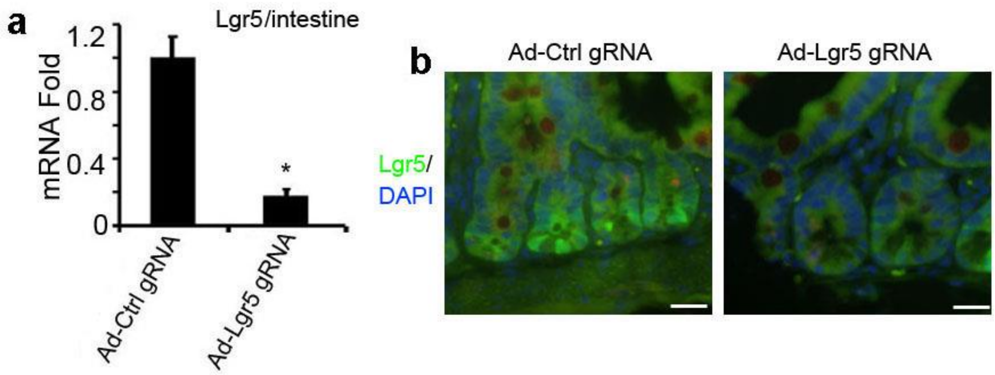


**Supplementary Figure 12:** Adenoviral delivery of CRISPR/CAS9 targeting LGR5 reduced Lgr5$^{+}$ intestine stem cells number. **a**. Adenovirus encoding CRISPR/CAS9 targeting LGR5 were injected into 8 week-old *Lgr5-GFP* mice i.v., the intestine crypt were collected at day 7 after virus injection, the mRNA level of Lgr5 were analysis using qPCR assay. **B.** Anti-GFP antibody was used to stain the Lgr5+ intestine cells 7 days after injection of adenovirus encoding CRISPR/CAS9 targeting LGR5 into 8 week-old *Lgr5-GFP* mice i.v.. n= 5 mice/group; for **a**, triplicates for each condition were analysed. Results are shown as mean ± s.d. of three independent experiments. *, $p$<0.05. Scale bar, 20 $\mu$m.

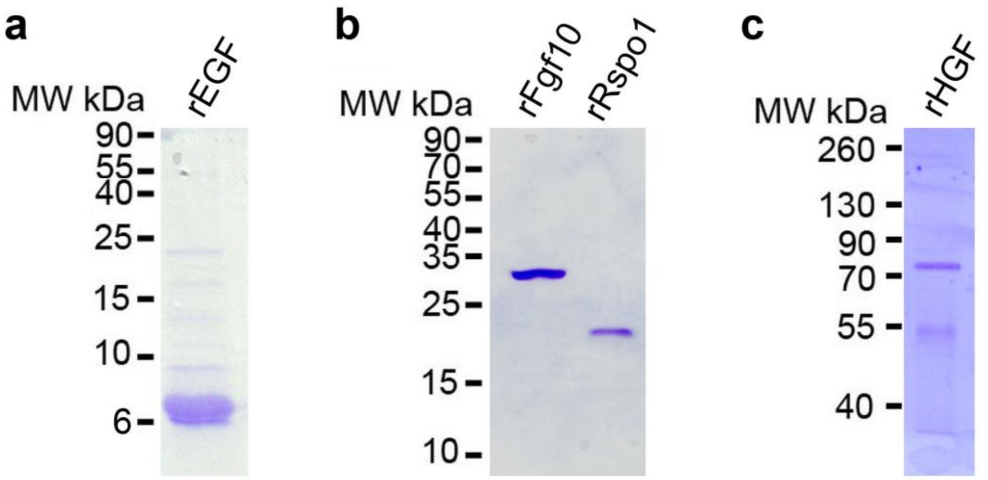


**Supplementary Figure 13: Purification of rEGF, rFgf10, rRspo1 and rHGF.**

Coomassie blue staining of purified rEGF (**a**), rFgf10 (**b**), rRspo1 (b) and rHGF (**c**), run under-non-reducing conditions. Results are representative of three or more separate protein preparations.

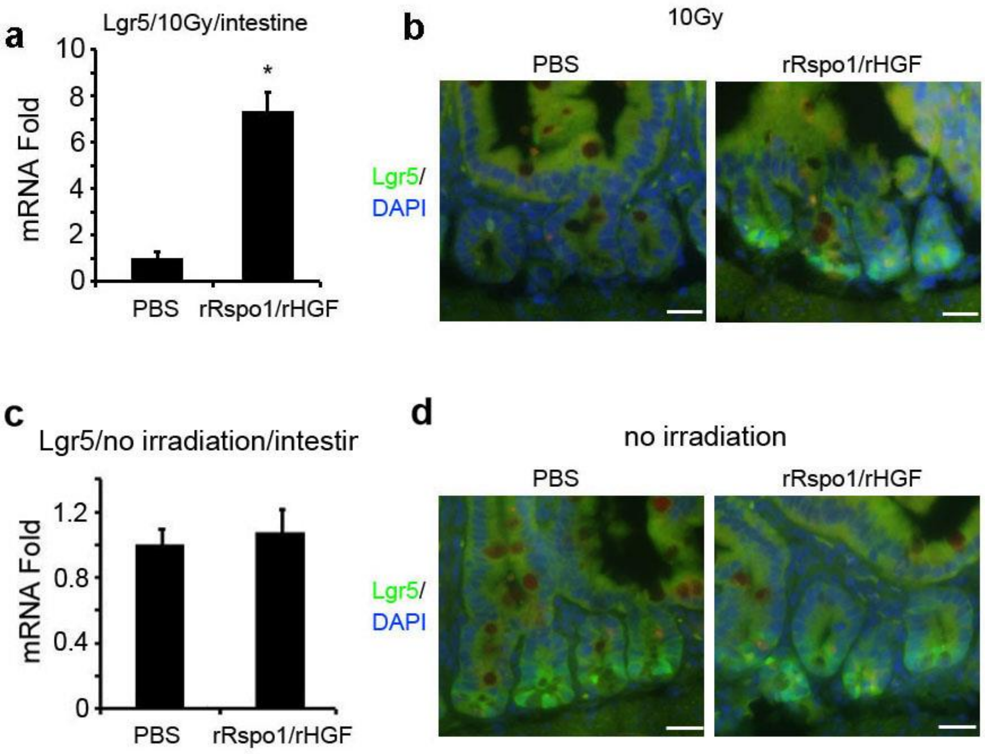


**Supplementary Figure 14: rHGF/rRspo1 induces Lgr5$^{+}$ intestine stem cells upon irradiation damage.** 8-week old Lgr5-GFP mice were treated with 10Gy irradiation, the mice were injected with rHGF/rRspo1 for three days (0.1 μg/mice/time/day). **a**. the mRNA level of Lgr5 were analysis using qPCR assay. **b.** Anti-GFP antibody was used to stain the Lgr5+ intestine cells. n= 5 mice/group. For **a**, **c**, triplicates for each condition were analysed. Results are shown as mean ± s.d. of three independent experiments. *, $p$<0.05. Scale bar, 20 μm (**c, d**).

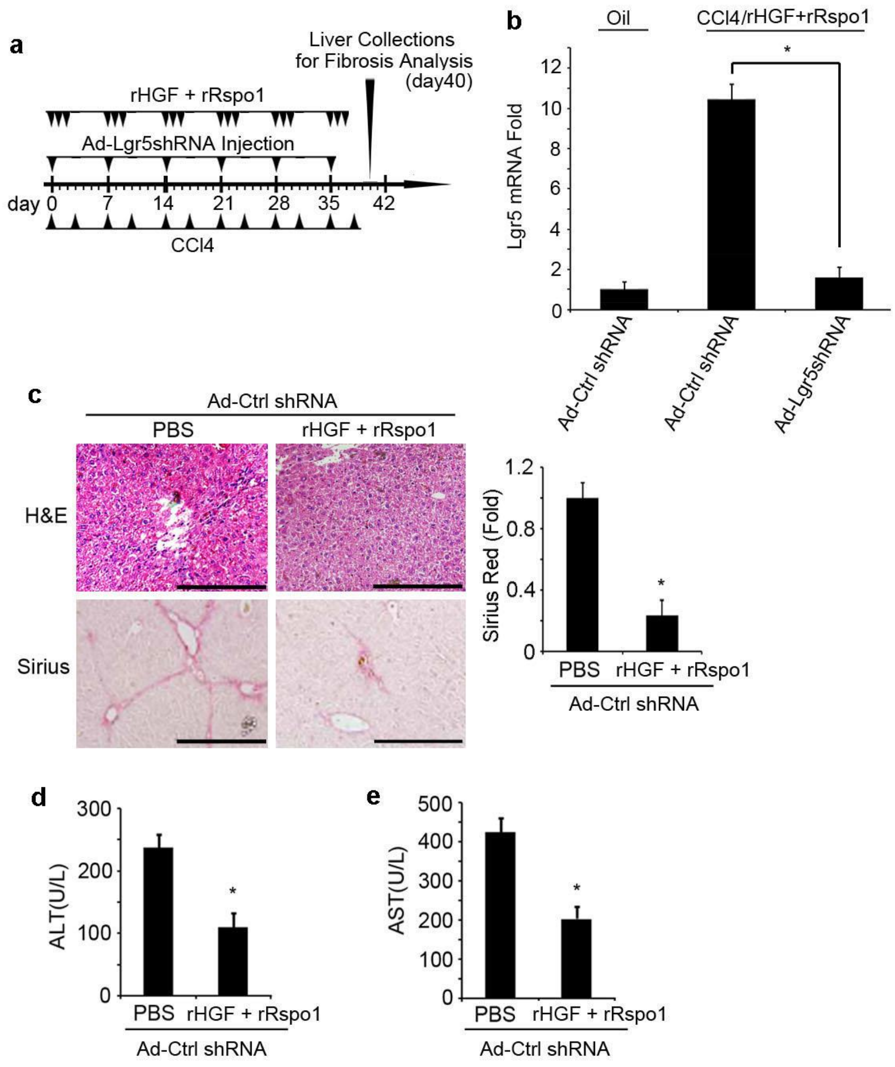


**Supplementary Figure 15: rHGF/rRspo1 decreased liver fibrosis when mice were infected with Ad-Ctrl shRNA. a,** Schematic overview of experimental setup. 8-week old C57BL/6J mice were i.p. injected with CCL4 (2ml/kg, Sigma-Aldrich) dissolved in olive oil at 1:4, or olive oil alone (2ml/kg) twice a week for 6 weeks. Ad-Lgr5shRNA or none sense control Ad-Ctrl shRNA control ($1x10^8$ pfu/mice) were injected into mice tail once every week for 6 weeks. rHGF plus rRspo1 proteins (0.1 μg/mice) or PBS control were injected into mice tail three times every week for 6 weeks. rHGF/rRspo1 decreased CCL4-induced liver Lgr5 mRNA level using qPCR assay (b), mitigated CCL4-induced liver fibrosis stained using H&E and Sirius red (c). Recovered liver functions by ALT and AST analysis (d, e). n= 6 mice/group. For **b, d, e**, triplicates for each condition were analysed. Results are shown as mean ± s.d. of three independent experiments. *, $p$<0.05. For **c**, Scale bar, 20 μm. Results are shown as mean ± s.d. of 5 independent sections taken randomly per mice and total 30 tissue specimens in each group (n=6 mice) *, $p$<0.05.

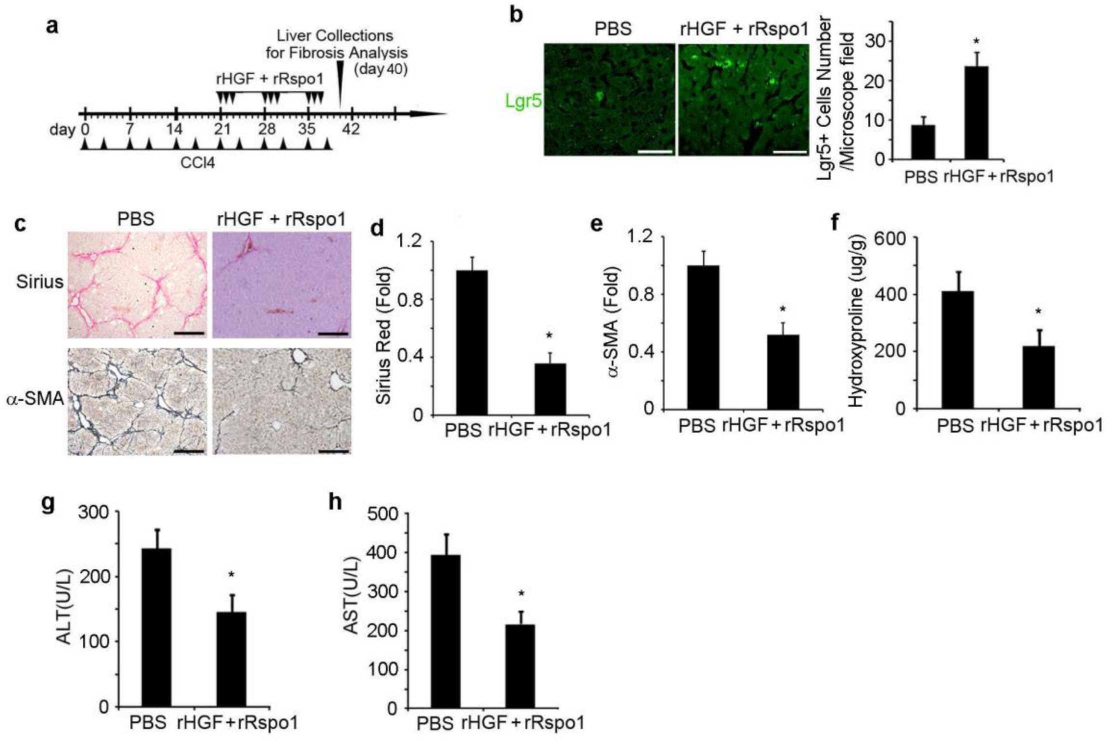


**Supplementary Figure 16. Therapeutic effect of rHGF/rRspo1 on Liver Fibrosis. a.** Schematic outline of therapeutic liver fibrosis model for b-h. 8-week old Lgr5-GFP mice were given CCL4 i.p. Twice weekly for 3 weeks, then rHGF/rRspo1 (0.1 μg/mice/time) or PBS control were inserted three times weekly, followed by a further 3 weeks of CCL4 i.p. twice weekly. The livers were harvested at day 40. **b,** Lgr5 expression was measured using Immunofluorescent assay with anti-GFP antibody . **c**, Sirius red (top) and α-SMA immunohistochemistry (bottom) of liver tissue for fibrosis analysis. **d**, Digital image analysis of sirius red staining in the therapeutic liver fibrosis model. **e**, Digital image analysis of α-SMA staining in the therapeutic liver fibrosis model. **f**, Hydroxyproline analysis of liver tissue in the therapeutic liver fibrosis model. **g, h**, The serum were harvested for ALT and AST analysis in the therapeutic liver fibrosis model. n=10 mice/group. Scale bar, 200 μm (b, c). For **b, d, e**, results are shown as mean ± s.d. of 5 independent sections taken randomly per mice and total 50 tissue specimens in each group (n=10 mice) *, $p$<0.05. For **f, g, h,** triplicates for each condition were analysed. Results are shown as mean ± s.d. of three independent experiments. *, $p$<0.05.

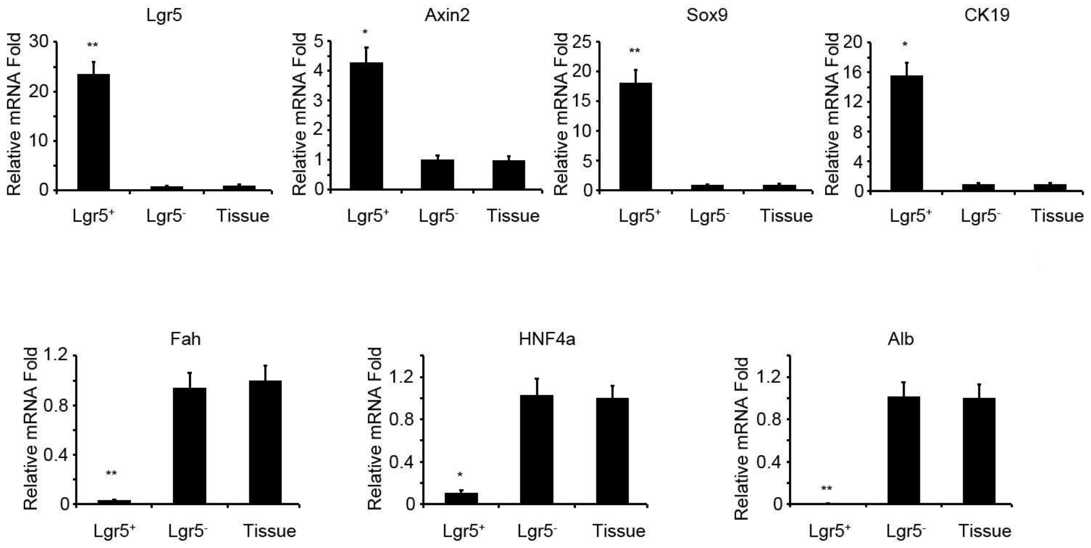


**Supplementary Figure 17: Expression of duct-like progenitor cells markers and hepatic markers in human Lgr5+ liver cells.** Total RNA was extracted from the human Lgr5+ liver cells sorted from the patents with liver fibrosis. These were reverse transcribed, PCR amplified and normalized to an endogeneous b-catenin control. Triplicates for each condition were analysed. Results are shown as mean ± s.d. of three independent experiments. *, $p$<0.05, **, $p$<0.01.

**Supplementary Table 1**: The effect sizes of each anti-fibrotic agent in reported studies and the general details of the experimental approach

| **Agents** | **Effect Sizes** **(Prophylactic or Therapeutic)** | **General details of the experimental approach** | **References** |
|---|---|---|---|
| SPARC | **Prophylactic**: The percentage of liver fibrotic area determined by image analysis was decreased in AdasSPARC-treated animals(0.11±0.04%) compared with Adβgal control (0.27±0.15%). **(around 30% reduction)** | Advanced liver fibrosis was induced in Sprague-Dawley rats by prolonged intraperitoneal administration of thioacetamide. 5 × $10^9$ TCID50 particles of adenovirus carrying antisense SPARC delivery via the tail vein, and directly into the liver 1 week after the first dose. Samples were harvested at week 7. | Adenovirus-mediated inhibition of SPARC attenuates liver fibrosis in rats. J Gene Med. 2008;10(9):993-1004.doi:10.1002/jgm.1228. |
| relaxin | **Therapeutic:** The percentage of liver fibrotic area significantly decreased at week 3 in rats infected with RLXexpressing adenovirus. **(around 60% reduction)** | Rats were given thioacetamide (TAA) for 8 weeks and infected once with either RLX-expressing adenovirus (1 × $10^9$ pfu per rat) via the tail vein. They were sacrificed 3 weeks after adenovirus infection. | A single adenovirus-mediated relaxin delivery attenuates established liver fibrosis in rats. J Gene Med. 2016 ;18(1-3):16-26. |
| BMP-7 | **Ref.1**: **Prophylactic**: The degree of collagen deposition in group with BMP7 treatment was lower than that in control group (week 9: 22.95 ± 6.66 vs 34.43 ± 6.96; | **Ref.1**: The mice were abdominally infected with S. japoni cum cercariae to induce a schistosomal hepatic fibrosis model, and were administered human BMP-7(300pg/g i.p., every | **Ref.1**: Exogenous bone morphogenetic protein-7 reduces hepatic fibrosis in *Schistosoma japonicum*-infected mice *via* |

| | | | |
|---|---|---|---|
| | week 15: 12.84 ± 4.36 vs 18.90 ± 5.07; $P < 0.05$) at both time points.<br><br>(**around 30% reduction**) | other day for a period of four weeks). Liver samples were extracted from mice sacrificed at 9 and 15 wk after modeling. Hepatic histopathological changes were assessed using Masson's staining. | transforming growth factor-b/Smad signaling.World J Gastroenterol. Mar 7, 2013; 19(9): 1405-1415. |
| | **Ref.2:** Therapeutic: Masson's staining for the BMP-7 +CCl4 group had less bridging fibrosis and collagen compared to the CCL4 group.<br><br>(**around 20% reduction**). | **Ref.2**: The model of liver fibrosis was induced by intraperitoneal injection with CCl4 three times per week lasting for 12 weeks in CCl4 group and the BMP-7+CCl4 group. After 8 weeks injection with CCl4, mice were intraperitoneal injected with human recombinant BMP-7 (300 pg/g body weight) in BMP-7+CCl4 group. Meanwhile, mice in the CCl4 group were only intraperitoneal injection with equal amount of saline. The degree of liver fibrosis was assessed by HE and Masson's staining. | **Ref.2**: BMP-7 attenuates liver fibrosis via regulation of epidermal growth factor receptor.Int J Clin Exp Pathol 2014;7(7):3537-3547 |

| | | | |
|---|---|---|---|
| | **Ref.3**: **Therapeutic:** A single administration of Ad-CAG-NCre and Ad-LNL-BMP-7 via the tail vein 4 weeks after the initiation of TAA injection considerably reduced the extent of fibrosis, as confirmed by morphometric analysis of Sirius red-stained liver sections.<br><br>(**around 30% reduction**) | **Ref.3**: Rats were treated with 200 mg/kg body weight of TAA twice per week for 4 weeks and were then injected with 2.06109 PFU of CAG-Cre and LNL-BMP-7 via the tail vein. TAA injection was continued for the next 3 weeks and animals were then sacrificed. | **Ref.3:** Adenovirus-mediated expression of BMP-7 suppresses the development of liver fibrosis in rats. Gut. 2007 May;56(5):706-14. Epub 2006 Nov 24 |
| HGF | **Ref.1:** **Therapeutic:** Moderate bridging fibrosis was observed in the livers of AAV5-HGF-transduced mice up to 6 weeks after transduction. However, AAV5-HGF markedly attenuated fibrosis at 9 or 12 weeks after transduction compared with control vector. Quantitative analysis of the fibrosis by image analysis showed a 50% reduction in fibrosis after AAV5-HGF transduction. (**around 50% reduction**). | **Ref.1:** A model of hepatic fibrosis was established by carbon tetrachloride (CCl4) administration in Balb/c mice. After the establishment of liver fibrosis,Adeno-associated virus (AAV) AAV5-HGF was injected once into the portal vein ($10^{11}$ vector genomes were transfected into the portal vein through the splenic hilum.). Mice were killed 3, 6, 9, and 12 weeks after injection. Another model was established by bile duct ligation (BDL). Seven weeks after AAV5-HGF injection, mice underwent BDL, and were then killed 2 weeks after BDL. | **Ref.1:** Adeno-associated virus vector-mediated production of hepatocyte growth factor attenuates liver fibrosis in mice<br><br>Hepatol Int (2008) 2:80–88 DOI 10.1007/s12072-007-9042-1. |

| | | | |
|---|---|---|---|
| | **Ref.2:** **Therapeutic:** Fibrosis was attenuated in livers transfected with the human HGF plasmid up to 14 days after HGF plasmid transfection , However , there was no significant fibrosis reduction at 21 days after HGF plasmid transfection. (**around 50% reduction at 14 days and no effect at 21 days**) | **Ref.2:** Naked plasmid DNA encoding human HGF (200 μg) was injected once, together with a hypertonic solution, into the hepatic artery after DMN treatment on three consecutive days per week for 3 weeks. | **Ref.2:** Hepatocyte growth factor gene transfer with naked plasmid DNA ameliorates dimethylnitrosamine-induced liver fibrosis in rats.Hepatology Research 2008; 38: 930–939 doi: 10.1111/j.1872-034X.2008.00340.x |
| uPA | **Therapeutic:** Application of 6 $10^{11}$ vp/kg AdHuPA adenovirus vector resulted in 25.8% liver fibrosis reduction. **(around 25% reduction)** | A biliary common duct ligation (BDL) rat model was used, rats ligated for 2 weeks and then, one single injection of 6 $10^{11}$ viral particles (vp)/kg clinical grade AdHuPA adenoviral vector was injected into mice | Improved Effects of Viral Gene Delivery of Human uPA plus Biliodigestive Anastomosis Induce Recovery from Experimental Biliary Cirrhosis. MOLECULAR THERAPY Vol. 9, No. 1, January 200430. |

| | | | |
|---|---|---|---|
| Smad7 | **Therapeutic:** Semiquantitative analysis of fibrotic areas 1 week after BDL displayed that collagen deposition was reduced to 43% after AdSmad7 infection in relation to those rats infected with AdLacZ. A similar Smad7-dependent effect was found in animals that were analyzed after 3 weeks of treatment （46 %）. **(around 45% reduction)** | Ligation of the common bile duct (BDL) was used to induce liver fibrosis in rats. Animals received injections of an adenovirus carrying Smad7 cDNA ( $1\ x10^{10}$ pfu) into the portal vein during surgery and via the tail vein at later stages. | Smad7 Prevents Activation of Hepatic Stellate Cells and Liver Fibrosis in Rats.GASTROENTEROLOGY 2003;125:178–191 |
| Bone marrow mesenchymal cells | **Ref.1:** **Therapeutic:** The amount of collagen deposition and alpha-SMA staining was about 40-50% lower in liver of rats with MSCs than that of rats without MSCs.<br><br>**(around 40-50% reduction)** | MSCs isolated from BM in male Fischer 344 rats were infused to female Wistar rats induced with carbon tetrachloride (CCl4) or dimethylnitrosamine (DMN). After 4-6 wk of MSCs administration, all rats were killed and fibrosis index were assessed by histopathology and radioimmunoassay. | Bone marrow-derived mesenchymal stem cells protect against experimental liver fibrosis in rats. World J Gastroenterol 2005;11(22):3431-3440. |
| | **Ref.2:** **Therapeutic:** Differences in fibrosis quantification were not found between MSC and placebo groups.<br><br>**(No effect)** | Ref.2: Female Wistar rats were fed exclusively an alcoholic liquid diet and received intraperitoneal injections of carbon tetrachloride every other day during 15 weeks. After this period, eight animals (MSC group) had 1 $10^{7}$ cells injected into the portal vein | **Ref.2:** Bone Marrow Multipotent Mesenchymal Stromal Cells Do Not Reduce Fibrosis or Improve Function in a Rat Model of Severe Chronic |

| | | | |
|---|---|---|---|
| | | while six animals (placebo group) received vehicle. Two months after cell delivery, animals were sacrificedfor analysis. | Liver Injury. STEM CELLS2008;26:1307–1314 |
| | **Ref.3:** α-SMA staining levels in intravenous- and intrahepatic-injected mice were significantly lower. **(around 50% reduction)** | **Ref.3:** MSCs isolated from Sprague Dawley (SD) rats were induced into hepatocyte-like cells . Liver fibrosis in SD rats was induced with carbon tetrachloride. Following hepatocyte induction in vitro, MSCs were transplanted by intravenous, intrahepatic, and intraperitoneal injection ($10^7$ cells/mL, and every rat was injected with 300 μL).Rats were sacrificed at 28 d post-implantation. | **Ref.3:** Intravenous injection of mesenchymal stem cells is effective in treating liver fibrosis.World J Gastroenterol 2012 March 14; 18(10): 1048-1058 |
| Rspo1 + HGF | **Prophylactic**: rHGF plus rRspo1 significantly reduced the fibrotic area in the CCL4-induced mice. **(around 75% reduction).** | rHGF and rRspo1 were injected at a dose of 0.1 g/mice respectively into the CCL4-treated C57 mice, three times a week for 6 week. | **Our current report** |

| | | | |
|---|---|---|---|
| | **Therapeutic:** rHGF/rRspo1significantly reduced liver fibrosis even after fibrotic disease had been established, as determined by collagen (sirius red) and a-SMA staining and hydroxyproline content. **(around 60% reduction)** | Following the treatment regimen as Dean Sheppard's lab did (*21*), we treated Lgr5-GFP mice with CCL4 for 3 weeks to establish fibrotic disease and then with rHGF/rRspo1 or PBS for the final 3 weeks of CCL4 | **Our current report** |
| Lgr5+ liver stem cells | **Prophylactic**: $10^5$ $Lgr5^+$ liver stem cells transplantation reduced the fibrotic area and decreased serum ALT and AST level significantly in the CCL4-induced mice. **(around 70% reduction).** | We isolated Lgr5-$GFP^+$ liver stem cells from *Lgr5-GFP* mice treated with 1XCCL4 by FACS, and injected these cells intrasplenically into the wild type C57 mice with acute damage (single CCL4 treatment) or chronic damage (liver fibrosis model, 2XCCL4 treatment/week for 6 weeks). | **Our current report** |

**Supplementary Table 2**: Age and gender of the 42 patients with clinically diagnosed liver fibrosis

| Patients Number | Age | Gender | Patients Number | Age | Gender |
|---|---|---|---|---|---|
| 1 | 22 | Male | 22 | 43 | Male |
| 2 | 25 | Male | 23 | 44 | Male |
| 3 | 26 | Male | 24 | 45 | Female |
| 4 | 28 | Male | 25 | 45 | Female |
| 5 | 28 | Female | 26 | 46 | Male |
| 6 | 29 | Male | 27 | 46 | Female |
| 7 | 30 | Female | 28 | 47 | Male |
| 8 | 31 | Male | 29 | 47 | Male |
| 9 | 31 | Male | 30 | 47 | Female |
| 10 | 31 | Female | 31 | 48 | Male |
| 11 | 33 | Male | 32 | 50 | Female |
| 12 | 33 | Male | 33 | 51 | Male |
| 13 | 34 | Female | 34 | 52 | Male |
| 14 | 36 | Male | 35 | 52 | Female |
| 15 | 36 | Male | 36 | 54 | Male |
| 16 | 37 | Male | 37 | 55 | Male |
| 17 | 38 | Female | 38 | 55 | Male |
| 18 | 40 | Male | 39 | 56 | Female |
| 19 | 41 | Male | 40 | 61 | Male |
| 20 | 41 | Male | 41 | 61 | Male |
| 21 | 42 | Female | 42 | 66 | Female |

**Supplementary Table 3**: Age and gender of the 5 automobile accident victims

| Patients Number | Age | Gender |
|---|---|---|
| 1 | 25 | Male |
| 2 | 28 | Female |
| 3 | 33 | Male |
| 4 | 36 | Male |
| 5 | 42 | Female |
